# Involvement of Human Volunteers in the Development and Evaluation of Wearable Devices Designed to Improve Medication Adherence: A Scoping Review

**DOI:** 10.3390/s23073597

**Published:** 2023-03-30

**Authors:** Lívia Luize Marengo, Silvio Barberato-Filho

**Affiliations:** Graduate Program in Pharmaceutical Sciences, University of Sorocaba, Sorocaba 18023-000, SP, Brazil; livia@liviamarengo.com.br

**Keywords:** digital technology, mobile health technology, wearable electronic devices, medication adherence, user-centered design, human volunteers

## Abstract

Wearable devices designed to improve medication adherence can emit audible and vibrating alerts or send text messages to users. However, there is little information on the validation of these technologies. The aim of this scoping review was to investigate the involvement of human volunteers in the development and evaluation of wearable devices. A literature search was conducted using six databases (MEDLINE, Embase, Scopus, CINAHL, PsycInfo, and Web of Science) up to March 2020. A total of 7087 records were identified, and nine studies were included. The wearable technologies most investigated were smartwatches (*n* = 3), patches (*n* = 3), wristbands (*n* = 2), and neckwear (*n* = 1). The studies involving human volunteers were categorized into idea validation (*n* = 4); prototype validation (*n* = 5); and product validation (*n* = 1). One of them involved human volunteers in idea and prototype validation. A total of 782 participants, ranging from 6 to 252, were included. Only five articles reported prior approval by a research ethics committee. Most studies revealed fragile methodological designs, a lack of a control group, a small number of volunteers, and a short follow-up time. Product validation is essential for regulatory approval and encompasses the assessment of the effectiveness, safety, and performance of a wearable device. Studies with greater methodological rigor and the involvement of human volunteers can contribute to the improvement of the process before making them available on the market.

## 1. Introduction

Wearable devices include watches, bracelets, stickers, textiles, and other accessories worn on the body, incorporating sensors and/or software linked to smartphones or tablets that can remotely collect continuous health-related information, actively or passively [1]. This equipment can integrate technology and computerized elements into clothing or communication devices attached to the body [2] and includes ingestible and implantable devices [3].

These devices can provide continuous access to real-time data, such as heart rate, blood glucose, sleep time, presence of airborne pathogens, respiratory anomalies, and drug concentration, allowing the tracking of patients’ health conditions without the need for frequent visits to medical centers or hospitals, as well as providing instant alerts in critical situations [4].

Wearable devices, in general, monitor physiological data through sensors performing clinical diagnoses by measuring different fluids, such as blood, saliva, tears and sweat, the main metabolites, ions, acids, electrolytes, heavy metals, alcohol, and toxic gases [5]. Sensors measure biological or chemical reactions, generating signals proportional to the concentration of an analyte in the reaction. Each sensor has optimized properties that are reflected in performance, such as selectivity, reproducibility, stability, sensitivity, and linearity [6].

Adherence to medication relies on the collaborative relationship between patients and health professionals in making decisions about medication treatment and personal behavior (mainly recognizing their willingness to participate) according to agreed recommendations [7].

An estimated 20–50% of all medication prescriptions for patients with chronic diseases are not in compliance with guidelines, resulting in non-adherence to medication [8].

Non-adherence to medication also occurs when a prescribed treatment is not started or is interrupted and when administration occurs at different amounts, times, or intervals from those prescribed, which can result in clinical worsening and additional costs to health systems [9,10].

With the aim of improving medication adherence, wearable devices are used to emit audible alarms, vibrations, or alert lights directly to users or their smartphones/tablets to indicate the time to administer the prescribed dose, identify and record the opening of medication bottles, and recognize the swallowing of the medication, among other functions [7,11].

Other alternatives for drug delivery in the promotion of medication adherence in patients with chronic diseases are wearable, implantable, and combined wearable and implantable devices. These new technologies have recently emerged for the treatment of patients with chronic diseases that require repeated and long-term medical attention, such as diabetes, eye diseases, cancer, wound healing, cardiovascular disease, and contraception [12].

The development of wearable devices includes the stages of conception and validation of the idea, followed by the construction and validation of the prototype (or minimum viable product), and finally, the product validation stage. The parameters most frequently evaluated include functionality, usability, acceptability, adherence to the device, and user satisfaction [13].

User experience tests are becoming the gold standard for determining technology adoption and analyzing user expectations involving the emotions underlying human–machine interactions. These tests also consider the user’s profile, including socioeconomic status, demographics, and needs or wants to be addressed by using the device [14].

In addition to user expectations, when deploying technology for human health, efficacy and safety tests should be carried out by conducting clinical studies (or trials) in which individuals volunteer and researchers take responsibility for the risks of the study, committing to reveal all clinical data, including unfavorable outcomes [15].

To be reliable, clinical trials must be methodologically robust, described in a clear and detailed protocol, and conducted in accordance with these procedures after having received prior approval or permit from a research ethics committee or an independent ethics committee [16]. When developing mobile and digital health interventions involving potential users from conception to product evaluation, this ensures the development and review of the content, structure, and flow of the intervention, identification of the best digital platforms for the target audience, and user understanding of the applications, purposes, and risks [17].

In 2019, an estimated 2000 patents involving wearable devices in medicine were granted in the United States and Europe, with 7500 in Asia [18]. The size of the global wearable medical device market is expected to reach USD 85.6 billion by 2027 [19].

Despite the progress and growth in the development of wearable devices to improve medication adherence, many factors need to be addressed to ensure the desired outcomes. Scoping reviews, a type of knowledge synthesis, follow a systematic approach to map evidence on a topic and identify the main concepts, theories, sources, and knowledge gaps [20].

In this context, the aim of this study was to investigate the involvement of human volunteers in the development and evaluation of wearable devices designed to improve medication adherence in the ideation, prototyping, and product testing phases. Such findings can provide relevant information for the scientific community, developers, and health professionals involved in the design and evaluation of wearable devices.

## 2. Method

### 2.1. Study Design

A scoping review study was conducted in accordance with the PRISMA Extension for Scoping Reviews (PRISMA-ScR): Checklist and Explanation [20].

### 2.2. Inclusion Criteria

Studies describing the involvement of human volunteers in the development and evaluation of wearable devices designed to improve medication adherence were included.

### 2.3. Exclusion Criteria

Studies not involving the use of medicines (such as monitoring vital signs, blood glucose, arterial hypertension, temperature, and other clinical parameters, monitoring of hearing, physical activity, mental disorders, etc.) were excluded.

Letters to the editors, systematic review protocols, narrative reviews, and conference abstracts were also excluded.

### 2.4. Data Sources and Search Period

A search for studies reporting the development and evaluation of wearable devices to improve medication adherence published up to 31 March 2020, with no restriction on date, language, or publication status, held on the MEDLINE (via PubMed), Embase (Excerpta Medica dataBASE), Scopus, CINAHL (Cumulative Index to Nursing and Allied Health Literature), PsycInfo, and WoS (Web of Science) databases was carried out. The search strategy used for the PubMed database, which combined MeSH terms and cross-referenced synonyms (entry terms) related to the main wearable technologies and medication adherence, is shown in Table 1. This same strategy was adapted for the other databases searched. Information from the gray literature was not included.

### 2.5. Study Selection

The Covidence^TM^ platform (https://www.covidence.org/home) was used to manage this step, allowing duplicate removal and record management.

Two reviewers independently assessed titles and abstracts to identify potentially eligible studies, advancing to full-text review when both reviewers confirmed inclusion. In the subsequent step, two reviewers also independently selected the studies that met the eligibility criteria by reviewing the full text of articles. Disagreements were resolved by consensus between reviewers in the two selection steps.

### 2.6. Data Extraction

A standardized pre-tested data extraction form (Microsoft Excel^TM^ 2019 MSO) with filling instructions was used. Data extraction was performed by the first reviewer (LLM), and the information obtained was later checked by the second reviewer (SB-F). Disagreements were resolved by consensus. When necessary, the authors were contacted for additional information.

The following data were extracted and grouped according to the device development stage: author(s); year of publication; country/countries where the survey was carried out; wearable technology type; methodological design; number of volunteers involved; prior approval by a research ethics committee; and results achieved.

### 2.7. Data Synthesis and Analysis

The included studies were classified based on the stages of wearable device development and the purpose of the trials, such as:

- Idea validation: also called the ideation phase, during which ideas are collected that serve to answer questions on the challenges identified and possible solutions. Concept validation or idea chaining can entail different research approaches, including interviews, observations, and behavioral mapping of potential users [21];
- Prototyping validation: also called the prototyping phase. This aims to provide physical means for experimentation and encourages early failure/success in the form of a test product at a reduced cost. It also serves as an object of transition during interdisciplinary collaboration and communication, as well as emphasizing the importance of the ability to visualize/manipulate solutions [22];
- Product validation: testing the product throughout the development phase reduces, or even eliminates, the chances of error and problems in the product under development [23]. In the case of medical devices, product testing that involves any investigation with humans aimed at discovering or verifying clinical effects is called a clinical trial [16].

The studies were also classified according to the type of technology:

- Smartwatches: these are digital watches that offer features such as heart rate monitoring, activity tracking, and providing reminders [24]. These watches rely on a compatible smartphone to deliver data over a Bluetooth^®^ connection and radio technology that provides solutions to meet specific connectivity needs [25]. As smartwatch apps can issue visual, verbal, audible, and vibrational alerts and reminders to wearers, they are useful for promoting medication adherence [26,27,28];
- Patches: these are thin, flexible, adhesive patch-like medical devices that use integrated circuits and nanomaterials to detect small amounts of toxins, proteins, DNA, or chemicals through the skin [29]. These wearable adhesive sensors can detect and record medication intake and emit vibrating signals at scheduled times for medication administration [30,31];
- Wristbands: these are equipped with sensors that can be used to monitor physical activity and the user’s heart rate and issue alerts for scheduled tasks. The bands also provide users with recommendations for health, fitness, and other warnings and can be programmed, for example, to receive reminders and notify the user when it is time to remove drugs to be administered from the bottles [32,33];
- Neckwear: these are devices that capture signs of swallowing and medication ingestion in the form of a necklace. They can also pair with mobile devices that receive and store data [11].

## 3. Results

**Figure 1:**
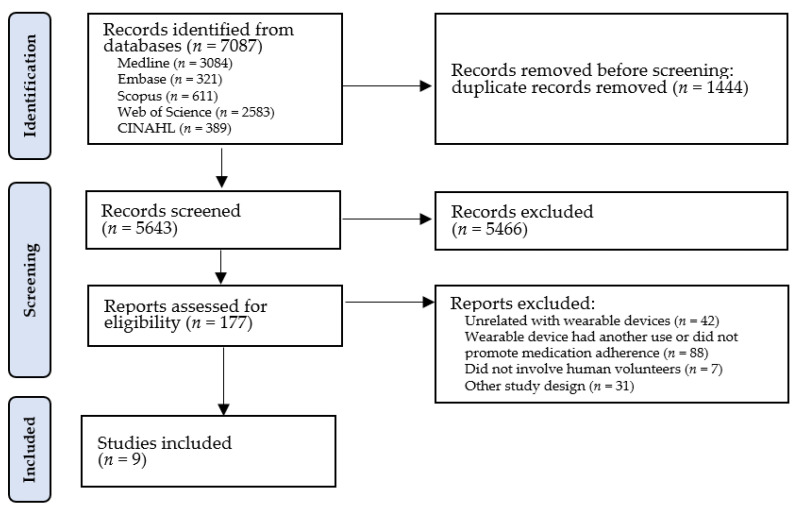
Flow diagram of study selection process based on PRISMA.

A total of 7087 records were identified in the databases consulted, from which 1444 duplicates were removed, giving a total of 5643 studies for title and abstract screening. This initial screening led to the exclusion of a further 5466 studies that failed to meet the eligibility criteria. After reading the full texts of the remaining 177 eligible studies, 161 were subsequently excluded for not meeting the eligibility criteria, leaving a total of 9 studies for inclusion in the review. The flow diagram in Figure 1 illustrates the study selection process.

The characteristics of the studies involving human volunteers in the validation of the idea, prototype, or product published between 2009 and 2019 included in the review are shown in Table 2. Wearable technologies aimed at improving medication adherence included smart watches (*n* = 3), adhesive sensors (*n* = 3), smart bracelets (*n* = 2), and smart necklaces (*n* = 1). Studies involving human volunteers were categorized based on the stages of wearable device development and the purpose of the trials: idea validation (*n* = 4); prototype validation (*n* = 5); or product validation (*n* = 1). One of the studies involving human volunteers validated both the idea and the prototype. Regarding the methodological design, surveys (*n* = 6), experimental studies (*n* = 3), and randomized clinical trials (*n* = 1) were identified. Only five articles reported prior approval by a local research ethics committee.

**Figure 2:**
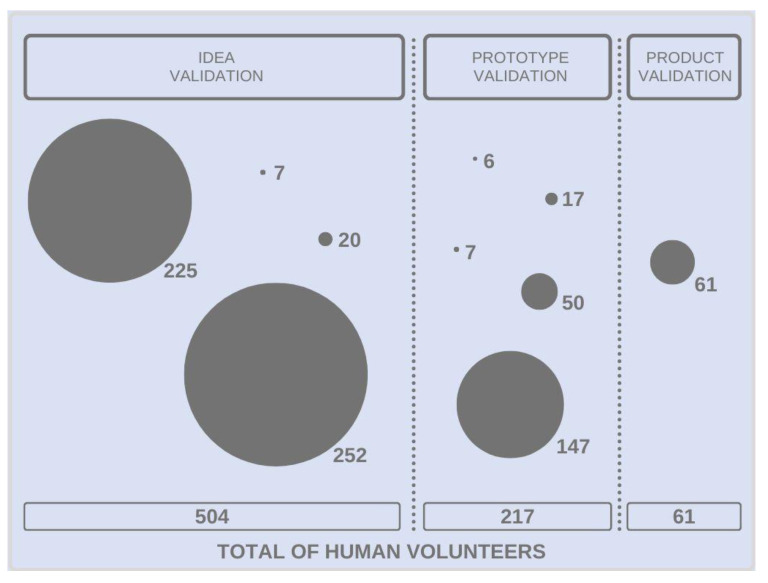
Number of human volunteers involved in idea, prototype, and product validation trials.

Figure 2 illustrates the number of human volunteers involved in each validation step: idea (*n* = 504), prototype (*n* = 217), and product (*n* = 61), totaling 782 participants.

### 3.1. Involvement of Human Volunteers in Idea Validation

Four studies involved human volunteers in idea validation.

Choi et al. (2013) [11] proposed a system in the form of a necklace and tablets labeled with radiofrequency identification (containing information on drug type, dose, manufacturer, expiration date, and serial number), with the aim of reminding patients when to administer and monitoring drug intake in real time. Twenty older people who used at least one medication for the treatment of chronic diseases were interviewed. The interviews aimed to understand the requirements of individual treatment regimens; understand how to administer medication; assess the usability and acceptability of the reminders used; gather opinions; and identify needs and concerns about the proposed technology. According to the authors, most users indicated that they liked the idea of a device that would help remind them to administer their medication, verify what they administered, and improve the possibility of connecting with their doctors and showed interest in the concept of the proposed device.

Rosner et al. (2015) [33] conducted an exploratory online survey of 252 respondents exploring potential user preferences for idea validation. The unavailability of the medication dose, administration at the wrong time, and interruption of treatment due to forgetfulness were the three main motivations for the development of a system of reminders and the monitoring of medication use. According to the authors, the results were promising for patients who administer medication at home and in hospitals, in addition to having been positively evaluated as a resource for caregivers and health teams.

Stekler et al. (2018) [34] investigated characteristics associated with adherence to identify which individuals would most benefit from the developed device (wrist sensor) and examined drug user practices and preferences, in addition to providing real-time reminders and information on adherence. A total of 225 participants were divided into two groups: 129 volunteers answered a self-assessment in person and periodically via the touch screens of tablets installed in the medical follow-up clinic. A second group of 96 volunteers, who were not part of clinical care, answered an anonymous online survey only once. The results allowed identification of the types of pill containers for which the system should be designed; volunteers’ interest in using a pulse sensor; and their preferences to receive reminders. According to the authors, the results supported the hypothesis and acceptance of wearable systems capable of detecting medication administration, providing reminders, and generating reports for users and healthcare providers.

Deutsch and Burgsteiner (2019) [35] investigated the possibilities and challenges in designing a smartwatch care system for older patients capable of recognizing emergencies through fall detection and inactivity recognition, as well as providing reminders for medication administration and obtaining assistance at the touch of a button. Seven healthy individuals participated in the tests, simulating walking, running, standing up, and sitting down, while the watch recorded their movements using sensors and transmitted this data to a smartphone. The information obtained was entered into the system, optimizing the ability to detect falls and inactivity (interruption of movement), enabling the user to manually request assistance, and developing reminders for medication administration. According to the authors, additional research is needed to evaluate and improve emergency detection capabilities through clinical studies, as well as to test the acceptance and usability of the system.

### 3.2. Involvement of Human Volunteers in Prototype Validation

Five studies involved human volunteers in prototype validation.

Abraham et al. (2013) [36] evaluated the ability to detect five vibration signals from a wearable device in the form of an adhesive patch. During a single experimental session, 50 volunteers were exposed to different intensities of vibratory signals. The current was gradually increased throughout the experiment. The volunteers indicated three transition points: (1) the beginning of signal perception; (2) when the signal was detectable enough to serve as a reminder; and (3) when that signal became uncomfortable. This feedback allowed the authors to identify an effective, discrete, and inaudible signal suitable for use in future testing and commercial versions of the device.

Subsequently, the same adhesive patch-type wearable device was evaluated by 167 volunteers for the detection capacity and acceptability of a set of fifteen continuous pulse vibration signals. The volunteers analyzed variables associated with the perception of reminder signals at three levels (very weak, appropriate, and very strong) and rated the safety of the tested device. For each signal detected, volunteers were asked to report adverse events, rate the acceptability of each signal (defined as “non-painful” or “painful”), and provide narrative comments about the signal [37].

According to the authors, most volunteers reported that (i) they would use the device as a reminder; (ii) they would recommend the device to others; (iii) they would prefer a signal of no more than 15 s; (iv) the proposed size was acceptable and they preferred the square shape; (v) they were willing to wear a patch for more than one day at a time; and (vi) they were satisfied or very satisfied with the privacy provided by the solution, its effectiveness as a reminder device, and that it would be unlikely to interfere with their daily routine of activities. The study allowed the authors to identify five effective and safe candidate signals for potential use in the wearable device. Regarding safety, nine adverse events were observed (in six volunteers): five involved small red rashes at the patch site; two were small red dots on the skin; one event was diaphoresis; and another was dizziness, blurred vision, and tinnitus [37].

After interviewing 252 volunteers in the validation of the idea, Rosner et al. (2015) [33] conducted an evaluation of the prototype by interviewing seven volunteers (four patients, two caregivers, and a member of the healthcare team) to evaluate the architecture of the system embedded in the smart bracelet developed. Volunteers answered questions about the device, features, and design. According to the authors, the volunteers’ opinions allowed reformulation of the system’s architecture, incorporating reliable and non-intrusive resources, functionalities, and alerts, which better matched patients’ needs.

In the study by Marquard et al. (2018) [38], 17 volunteers tested two prototypes of smart bracelets containing a motion detection sensor coupled to a placebo pill bottle and connected to a smartphone application, with the aim of understanding the usage practices of medicines and users’ technological preferences. Through face-to-face interviews, the authors investigated how the volunteers stored, remembered to administer, and removed their medicines from the vials. Volunteers independently completed an electronic questionnaire assessing their perceived levels of adherence, medication use practices, and preferences to obtain feedback on adherence patterns. Volunteers were more interested in receiving reminders through the wristband or a mobile app than through text messages. The authors planned to carry out a pilot validation study in which volunteers used the system for six months with the aim of improving the accuracy and efficiency of the sensors and algorithms for detecting the movement of opening the bottles (understood as drug administration).

Espinoza et al. (2009) [25] developed the prototype of a smartwatch incorporating emergency healthcare buttons and a notification screen to support medication adherence by older users. Six volunteers participated in the validation through interviews based on the Medication Management Instrument for Disabilities in the Elderly (MedMaIDE), which assesses possible problems involving medication adherence in the home environment [39]. According to the authors, the volunteers considered the system appropriate but suggested increasing the size of some interface elements.

### 3.3. Involvement of Human Volunteers in Product Validation

Only one study involved human volunteers in product validation.

The randomized controlled trial by Browne et al. (2019) [29] involved 61 volunteers undergoing tuberculosis treatment divided into two groups: the first group (*n* = 41) involved WOT (wirelessly observed therapy) with the use of a system for checking swallowing (consisting of three items: an ingestion sensor; a detector with an adhesive support worn on the torso; and a paired mobile device); the second group (*n* = 20) involved DOT (directly observed therapy) in which a health professional directly observed the swallowing of the medication and provided written verification of the adherence and completion of the treatment. The WOT system provided real-time reporting, supporting patient self-management and enabling rapid remote identification of those who needed more support to maintain adherence. According to the authors, in terms of accuracy, WOT was equivalent to DOT. In confirming daily adherence to medication during tuberculosis treatment, WOT was superior to DOT, and all volunteers preferred WOT despite the occurrence of some adverse events, such as skin rash and itching, associated with the patch.

## 4. Discussion

Regarding the studies reviewed, the involvement of human volunteers was found predominantly at the stages of idea and prototype validation. The methodological designs used in these stages included surveys to obtain information from users on their interest in wearable technology or guide developers in identifying the most convenient technical requirements according to the users’ perspectives and experimental studies involving tests to evaluate a prototype. The only study aimed at product validation was a randomized clinical trial.

According to Jiang, Mück, and Yetisen (2020) [40], to convert a wearable device into an innovation and make this a viable commercial product, strategies for manufacturing and regulatory approval processes should be implemented from the early stages of development.

Manufacturers need to understand the risks, timings, and costs associated with bringing a robust product to market given that, akin to medical devices, wearable technologies are also subject to regulation. Conducting rigorous preliminary testing typically takes 2–3 years and requires a financial investment of around USD 10–20 million. The Food and Drug Administration (FDA, Silver Spring, MD, USA) warns that most of the new medical devices will not achieve clinical and market approval [41].

Validation is critical to ensure that sensor recordings are accurate and sensitive enough for medical diagnosis and prognosis. This is crucial to ensure not only the generalizability of a sensor within a target population but also its ability to measure day-to-day variability data, which can be confirmed by disease symptoms. To this end, data collected from commercially available wearable sensors should be systematically compared to data acquired by reference medical devices (i.e., reliable gold standard systems, medical scores, or groups of subjects) [42].

In general, product validation involves qualitative research to explore areas such as values, preferences, acceptability, feasibility, and equity implications. This, in turn, involves users, focus groups, experimental studies, and clinical trials that should be planned and executed in the product development phase to investigate possible failures, propose improvements, meet regulatory requirements, and increase the chance of commercial success.

As with any study involving human volunteers, this work requires approval by a research ethics committee. However, of the nine studies included, only five (56%) reported this prior approval, covering the three methodological designs observed.

The most fitting method for evaluating digital health products depends on the desired objective and may involve descriptive, comparative, qualitative, and economic studies. Descriptive studies reveal the state of the art and can provide descriptive statistics or investigate correlations. Comparative studies verify whether the product or prototype works properly, collecting quantitative data and comparing it against an alternative, such as individuals who have not used digital technology. Qualitative studies, on the other hand, elucidate how users experience the product and collect their perceptions. Lastly, economic studies seek to estimate the relationship between the benefits of the product versus the cost implications for implementation [43].

To demonstrate effectiveness and value for digital technologies, standards that describe the level of evidence required for the different functions and risks of the technologies’ lifecycle, such as design, value description, performance demonstration, value delivery, and deployment, are required. These must meet standards of safety, incorporate acceptability to users, consider environmental sustainability, health inequalities and prejudice mitigation, incorporate good data practices, define levels of professional supervision, and elucidate processes for creating reliable health information [44].

Regarding the number of participants, the studies that involved the largest number of human volunteers were surveys, two of which were carried out online. It should be noted that studies with larger samples are more representative and contribute to obtaining more complete information.

Two studies reported adverse events in the trials performed, ranging from minor red rashes and itching at the patch site to diaphoresis, dizziness, blurred vision, and tinnitus. These observations confirm that wearable devices also pose a risk and that their use should be evaluated and monitored responsibly by manufacturers, healthcare professionals, and users.

The ease with which applications can be made available in specialized online stores (Google Play or Apple Store) favors access by users but lacks the more stringent regulation established for other medical devices. This less regulated space is attractive to developers, who typically seek market access as opposed to adoption by health systems, which would require a more thorough assessment of the risks and benefits.

A systematic review of diagnostic accuracy studies carried out by Freeman et al. (2021) [45] examined the accuracy of algorithm-based smartphone applications (“apps”) to assess the risk of skin cancer in suspicious skin lesions. All skin cancer smartphone apps based on evaluated algorithms disclaim liability, indicating that the results are to be used as a guide only and cannot replace health advice. Therefore, these apps try to evade any responsibility for the negative results experienced by users. Nevertheless, this review found poor and variable performance of algorithm-based smartphone apps, which indicates that these apps have not shown sufficient promise to recommend their use. Further, the American Federal Trade Commission has fined the marketers of two apps (MelApp and Mole Detective) for “deceptively claiming the apps accurately analyzed melanoma risk”.

In our scoping review, two studies, one at the idea validation stage and the other at the prototype validation stage, clearly addressed the need for additional research to assess signal detection capabilities, test system acceptance and usability, and improve the accuracy and efficiency of the sensors and algorithms. This reinforces the need for in-depth testing and validation of devices before making them available on the market.

Possible market barriers to the development and evaluation of wearable devices include the incipient regulation and disconnect between developers and healthcare professionals.

Considering that the development of wearable devices needs to be rapid, Farao et al. (2020) [46] highlighted that, even in development contexts with limited resources, combining the structure of a device with development based on user-centered holistic design allows rapid improvements throughout the development process.

The involvement of human volunteers in the development of technological products may involve concepts such as human-centered design (HCD), design thinking (DT), and user-centered design (UCD). A review by Göttgens and Oertelt-Prigione (2021) [47] summarizes the application of HCD practices across various areas of health innovation. All approaches prioritized the user’s needs and the participatory and iterative nature of the design process. The design processes comprised several design cycles during which multiple qualitative and quantitative methods were used in combination with specific design methods. The increasing use of design-based approaches, such as HCD, DT, and UCD in health research, subjects them to evaluation according to traditional biomedical standards. However, the analytical approach of the scientific method versus the constructive approach of the design method makes it impossible to evaluate both methods to the same standard. For the validation of design methods, a relativistic approach that increases confidence in the methods can be considered a more appropriate paradigm for design methods, particularly those that are concerned with the subjective elements of this process.

A multi-stakeholder workgroup from diverse backgrounds (hospital administration, clinical medicine, academia, insurance, and the commercial device industry) was convened by two of the National Institutes of Health’s Big Data to Knowledge (BD2K) Centers of Excellence—the Mobilize Center at Stanford University and the Mobile-Sensor-to-Knowledge Center (MD2K)—to evaluate the clinical application of wearables and identify common features responsible for their success. Seven features were identified, including a clearly defined problem, integration into a system of healthcare delivery, technology support, personalized experience, focus on end-user experience, alignment with reimbursement models, and the inclusion of clinician champions [48].

A strength of this scoping review is the comprehensive systematic search, covering six healthcare databases and peer review in screening, study selection, and data extraction. However, some relevant studies may not have been retrieved due to the wide variety of descriptors and cross-referencing synonyms used in the indexing of studies, the dynamism of the area, and publication in specific computer science or engineering journals. Given this was a scoping review, the methodological quality of the studies reviewed was not evaluated; therefore, any inferences regarding measures of effect should be interpreted with caution.

In addition to the need to carry out robust trials confirming the utility of wearable devices in promoting medication adherence, several questions should be addressed in future studies: What is the magnitude of the improvement in medication adherence obtained? What are the recommended study designs and follow-up timing for effectiveness and safety assessments? Are the benefits achieved with the use of wearable devices maintained over time? Is the technology affordable, and are the costs acceptable?

These knowledge gaps may guide further investigations that increase confidence in the recommendation and use of wearable devices to improve medication adherence.

## 5. Conclusions

Wearable devices have been successfully used in different clinical conditions. Through the continuous monitoring of medication use, they record and transmit data in real time and can issue alerts to users, caregivers, and/or health providers. This consistent follow-up, with immediate feedback, counseling, and guidance, represents a new paradigm in healthcare.

In the wearable device development cycle, although the involvement of human volunteers in all three phases (idea, prototype, and product validation) was evident, this participation was characterized by fragile methodological designs, a lack of a control group, a small number of volunteers, a short follow-up time, and studies which failed to report approval by an ethics committee.

Product validation is essential for regulatory approval and encompasses the assessment of the effectiveness, safety, and performance of a wearable device. Studies with greater methodological rigor and the involvement of human volunteers can contribute to the improvement of the process before making them available on the market.

## Figures and Tables

**Table 1:** Keywords and search strategy for the Medline database (PubMed).

Number	Search Strategy
#1	(wearable device) OR (wearable devices) OR (wearable electronic device) OR (wearable electronic devices) OR (wearable technologies) OR (wearable technology) OR (wearable health monitoring devices) OR (technologies wearable) OR (technology wearable) OR (device wearable) OR (devices wearable) OR (wearable wrist biosensor) OR (wearable*) OR smartwatch OR (smart watch) OR smartwatches OR (smart watches) OR wristband* OR (hearing aids) OR (hearing aid) OR (ear mold) OR (ear molds) OR (earmold) OR (earmolds) OR (electronic tattoo) OR (electronic tattoos) OR (optical tattoo) OR (optical tattoos) OR (head mounted display) OR (head mounted displays) OR (subcutaneous sensors) OR (subcutaneous sensor) OR (electronic footwear) OR (electronic textile) OR (wireless sensor) OR (body sensor) OR (body worn sensor) OR (electronic footwear) OR (electronic textiles) OR (wireless sensors) OR (body sensors) OR (body worn sensors) OR biosensor OR biosensors OR accelerometer* OR gyroscope* OR (optical sensor) OR (contact sensor) OR (optical sensors) OR (contact sensors) OR (wearable monitor) OR (wearable monitors) OR (chips diagnosis) OR (electronic skin)
#2	(medication adherence) OR (medication compliance) OR (medication non adherence) OR (medication nonadherence) OR (medication non-adherence) OR (medication noncompliance) OR (medication non-compliance) OR (medication persistence) OR (therapeutic adherence) OR (therapeutic adherence and compliance) OR (treatment adherence) OR (treatment adherence and compliance) OR (compliance patient) OR (patient adherence) OR (adherence patient) OR (patient cooperation) OR (cooperation patient) OR (patient non-compliance) OR (non-compliance patient) OR (patient non compliance) OR (patient nonadherence) OR (nonadherence patient) OR (patient noncompliance) OR (noncompliance patient) OR (patient non-adherence) OR (non-adherence patient) OR (patient non adherence) OR (treatment compliance) OR (compliance treatment) OR (treatment compliances) OR (therapeutic compliance) OR (compliance therapeutic) OR (compliances therapeutic) OR (therapeutic compliances)
#3	#1 AND #2

**Table 2:** Characteristics of studies included in review.

Author	Country of Origin	Type of Technology	Technology Description	Study Design	Ethics Committee and Informed Consent
Idea Validation					
Choi et al., 2013 [11]	USA	Neckwear	Neckwear device with a proposed system that reminds patients when to take their medications and the proper dose of each pill and monitors medication ingestion in real-time	Survey	Not reported
Rosner et al., 2015 * [33]	Romania	Wristband	Development of a medication reminder system that delivers alarms effectively through a user-sensitive design to be easily integrated into patients’ and caregivers’ daily routines	Survey	Not reported
Stekler et al., 2018 [34]	USA	Smartwatch	Wrist worn sensor using Bluetooth technology for motion sensing and gesture recognition, tags on medication bottles, a smartphone app, and real-time adherence reminders	Survey	Yes
Deustch; Burgsteiner, 2019 [35]	Austria	Smartwatch	Smartwatch-based assistance system which can set medication reminders and get help from relatives at the push of a single button	Experimental study	Not reported
Prototype Validation					
Espinoza et al., 2009 [25]	Mexico	Smartwatch	User interface for informing (coaching) older adults on the medications and doses to take	Survey	Not reported
Abraham et al., 2013 [36]	USA	Patch	Electronic skin patch designed to deliver discreet tactile reminder stimuli	Experimental study	Yes
Abraham et al., 2015 [37]	USA	Patch	Electronic skin patch designed to deliver discreet tactile reminder stimuli	Experimental study	Yes
Rosner et al., 2015 * [33]	Romania	Wristband	Development of a medication reminder system that delivers alarms effectively through a user-sensitive design to be easily integrated into patients’ and caregivers’ daily routines	Survey	Not reported
Marquard et al., 2018 [38]	USA	Wristband	Detection of pill-taking behavior, triggering pill-taking reminders for wrist wearers	Survey	Yes
Product Validation					
Browne et al., 2019 [29]	USA	Patch	Small adhesive-backed detector patch worn on the torso and a paired mobile device	Randomized controlled trial	Yes

Notes: USA—United States of America; * Idea and prototype validation in same study.

## Data Availability

The analyzed data can be seen in the studies cited in this scoping review. Data sharing is not applicable to this article.
